# Selective deletion of E3 ubiquitin ligase FBW7 in VE-cadherin-positive cells instigates diffuse large B-cell lymphoma in mice in vivo

**DOI:** 10.1038/s41419-024-06597-7

**Published:** 2024-03-14

**Authors:** Zhaohua Cai, Shaojin You, Zhixue Liu, Ping Song, Fujie Zhao, Junqing An, Ye Ding, Ben He, Ming-Hui Zou

**Affiliations:** 1 Department of Endocrinology and Metabolism, Tianjin Medical University General Hospital, 154 Anshan Road, Tianjin, 300052 China; 2 Department of Cardiology, Shanghai Chest Hospital, Shanghai Jiao Tong University School of Medicine, Shanghai, 200030 China

**Keywords:** Ubiquitin ligases, Cancer models

## Abstract

During the maturation of hematopoietic stem/progenitor cells (HSPCs) to fully differentiated mature B lymphocytes, developing lymphocytes may undergo malignant transformation and produce B-cell lymphomas. Emerging evidence shows that through the endothelial-hematopoietic transition, specialized endothelial cells called the hemogenic endothelium can differentiate into HSPCs. However, the contribution of genetic defects in hemogenic endothelial cells to B-cell lymphomagenesis has not yet been investigated. Here, we report that mice with endothelial cell-specific deletion of Fbw7 spontaneously developed diffuse large B-cell lymphoma (DLBCL) following Bcl6 accumulation. Using lineage tracing, we showed that B-cell lymphomas in Fbw7 knockout mice were hemogenic endothelium-derived. Mechanistically, we found that FBW7 directly interacted with Bcl6 and promoted its proteasomal degradation. FBW7 expression levels are inversely correlated with BCL6 expression. Additionally, pharmacological disruption of Bcl6 abolished Fbw7 deletion-induced B-cell lymphomagenesis. We conclude that selective deletion of E3 ubiquitin ligase FBW7 in VE-cadherin positive endothelial cells instigates diffuse large B-cell lymphoma via upregulation of BCL6 stability. In addition, the mice with endothelial cell-specific deletion of Fbw7 provide a valuable preclinical platform for in vivo development and evaluation of novel therapeutic interventions for the treatment of DLBCL.

## Introduction

The lymphomas remain a major public health challenge around the globe. They represent one of the most heterogeneous groups of malignancies in all of cancer medicine. Non-Hodgkin lymphoma (NHL) which accounts for 90% of all lymphomas consists of more than 60 subtypes [1]. B-cell non-Hodgkin lymphomas are also a large heterogeneous group of hematologic malignancies. Given that the vast majority of B-cell lymphomas arise from the malignant transformation of germinal-center or post-germinal-center B cells, it is remarkable that B-cell lymphomas occur in such numerous clinically and histopathologically distinct varieties. The variability of B-cell lymphomas has been attributed to the distinct stages of differentiation and various events during which tumor progenitors become transformed [2, 3]. Recent evidence regarding the diverse origins of hematopoietic stem/progenitor cells (HSPCs) [4–6] might also account for this high variability. Most notably, HSPCs are derived directly from a unique population of endothelial cells known as hemogenic endothelium through a process called endothelial-hematopoietic transition (EHT) [4, 5, 7]. However, the contribution of endothelial genetic defect to B-cell lymphomagenesis remains unknown.

FBW7 is an E3-ubiquitin protein ligase that is part of the SKP1-cullin-F-box complex and functions as a tumor suppressor by targeting oncoproteins for degradation. Deletion or mutation of FBW7 is strongly implicated in numerous human cancers, including T-cell and B-cell malignancies [8–12]. In addition, mice with hematopoietic cell-specific Fbw7 deletion develop T-cell acute lymphoblastic leukemia [13], while T-cell deletion of Fbw7 leads to thymic lymphoma [14]. Although there is emerging evidence indicating the potential role of FBW7 in diffuse large B-cell lymphoma (DLBCL) [15, 16], direct experimental evidence implicating FBW7 in DLBCL pathogenesis is still lacking.

BCL6 is the product of a proto-oncogene that is strongly implicated in the pathogenesis of DLBCL. In many DLBCL patients, deregulation of BCL6 expression is achieved through chromosomal rearrangement or somatic mutation of the 5′-noncoding regions of BCL6 [17, 18]. BCL6 has been demonstrated to be degraded by an SKP1-CUL1-F-box protein (SCF) ubiquitin ligase complex containing the F-box protein FBXO11, which is inactivated in DLBCLs. Moreover, Park et al. reported that the signal-responsive E3 ubiquitin pellino 1 promotes B-cell lymphomagenesis by deregulating BCL6 polyubiquitination [19]. Therefore, the posttranslational modifications that regulate the BCL6 stability may also play important roles in B-cell lymphomagenesis. However, whether or not BCL6 accumulation leads to DLBCL in vivo remains largely unknown.

In this present study, we found that the mice with endothelial cell-specific deletion of Fbw7 spontaneously developed DLBCL following Bcl6 accumulation. We demonstrated that B-cell lymphomas in Fbw7 knockout mice were hemogenic endothelium-derived. Mechanistically, we found that FBW7 directly interacted with BCL6 and promoted its proteasomal degradation. In addition, pharmacological disruption of Bcl6 abolished Fbw7 deletion-induced B-cell lymphomagenesis. These findings suggest that FBW7 plays an essential role in B-cell lymphomagenesis by controlling BCL6 ubiquitination and stability.

## Results

### Endothelial Fbw7 deficiency induces diffuse large B-cell lymphoma

**Fig. 1:**
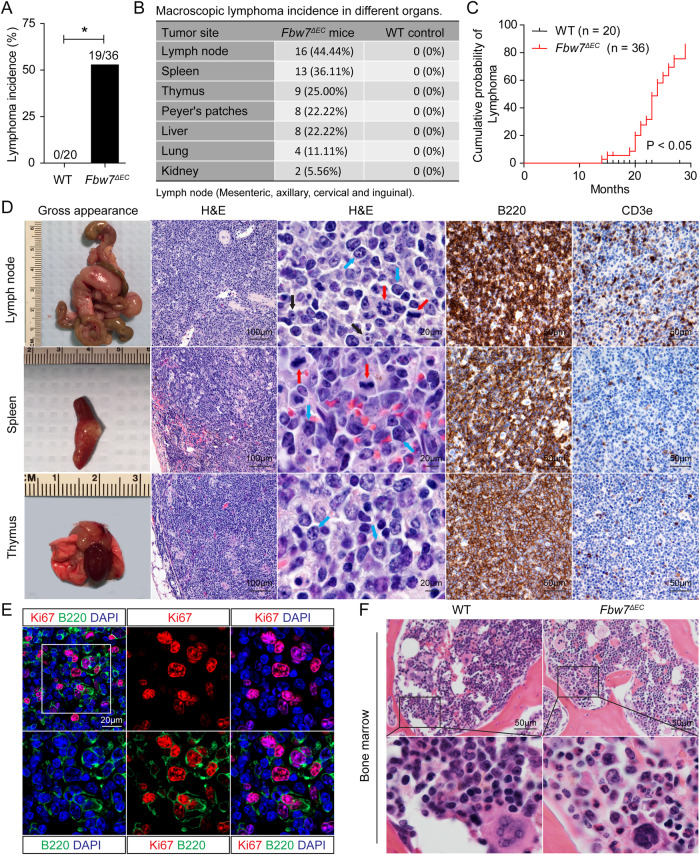
Fbw7 deficiency in endothelial cells induces diffuse large B-cell lymphoma in mice. **A** Lymphoma incidence in *Fbw7*^*ΔEC*^ (*n* = 36) and WT mice (*n* = 20). Statistics were determined using Fisher’s exact test. **B** Macroscopic lymphoma incidence in indicated organs. **C** Kaplan–Meier plot illustrating time to lymphoma development. Statistical analysis was performed using the log-rank test. **D** Macroscopic images, H&E, and immunohistochemistry analysis for B220 and CD3e in lymphoma tissue from indicated tissues. Blue and black arrows denote centroblasts and immunoblasts, respectively. Red arrows denote mitotic cells. **E** Immunofluorescence staining for B220 (green), Ki67 (red), and DAPI (blue) in lymphoma tissues from *Fbw7*^*ΔEC*^ mice. **F** H&E staining of bone marrow from WT and *Fbw7*^*ΔEC*^ mice.

To determine the possible contribution of endothelial genetic defect to lymphomagenesis, we generated mice with endothelial cell-specific Fbw7 deletion (*Fbw7*^*ΔEC*^; Figure S1A–C, Supporting Information). Starting at 14 months of age, we observed that 19/36 (52.78%) *Fbw7*^*ΔEC*^ mice spontaneously developed large neoplasms in multiple organs, including the lymph nodes (LNs), spleen, thymus, Peyer’s patches, liver, lungs, and kidneys; these neoplasms did not occur in age-matched wild-type (WT) littermates (Fig. 1A, B). After adjusting crude incidence for loss of individual mice to other causes, such as eosinophilic crystalline pneumonia, corneal plaques, and undiagnosed terminal illness, 83.69% of *Fbw7*^*ΔEC*^ mice spontaneously developed lymphoma by 29 months of age (Fig. 1C). These neoplasms in *Fbw7*^*ΔEC*^ mice exhibited partial or complete effacement of normal tissue architecture by diffuse infiltration of large centroblast-like or immunoblast-like cells with high-grade nuclear features, including prominent nucleoli, irregular nuclear contours, and frequent mitotic figures (Fig. 1D and Fig. S2, Supporting Information). Immunohistochemical analyses demonstrated that these lymphomas exhibited abundant expression of B-cell markers (B220 and CD20) and much weaker expression of the T-cell marker CD3e, confirming their B-cell origin (Fig. 1D and Figs. S2, S3, Supporting Information). Neoplastic cells exhibited robust proliferation and increased apoptosis, as illustrated by intense Ki67 staining, upregulation of proliferating cell nuclear antigen (PCNA), and cleaved caspase-3; these increases were accompanied by concomitant down-regulation of P27, P21, Bcl2, and Bclxl (Fig. S4, Supporting Information). Notably, Ki67 was predominantly expressed in large B cells (Fig. 1E). Together, these phenotypic characteristics strongly support the diagnosis of DLBCL in *Fbw7*^*ΔEC*^ mice. Moreover, peripheral blood cell counts from *Fbw7*^*ΔEC*^ and WT mice showed comparable levels of white blood cells, lymphocytes, and monocytes (Figs. S5, S6, Supporting Information), and bone marrow sections showed no obvious morphological abnormalities (Fig. 1F), further excluding the diagnosis of leukemia.

With a panel of clinically relevant markers for B-cell lymphoma, an immuno-pathological phenotyping analysis was conducted in 11 mice with DLBCL. Based upon the expression profiles of CD10, Bcl6, and MUM1 using Han’s algorithm [20], all cases were identified as the germinal center B-cell-like subtype of DLBCL (Table S1 and Fig. S7, Supporting Information). Specifically, Bcl6 was upregulated in all DLBCL cases (Table S1 and Fig. S7, Supporting Information). Additionally, the intensity of MUM1, Bcl6, c-Myc, and CD5, or the Ki67 index, was not correlated with the clinical stage (*P* > 0.05 for all comparisons).

**Fig. 2:**
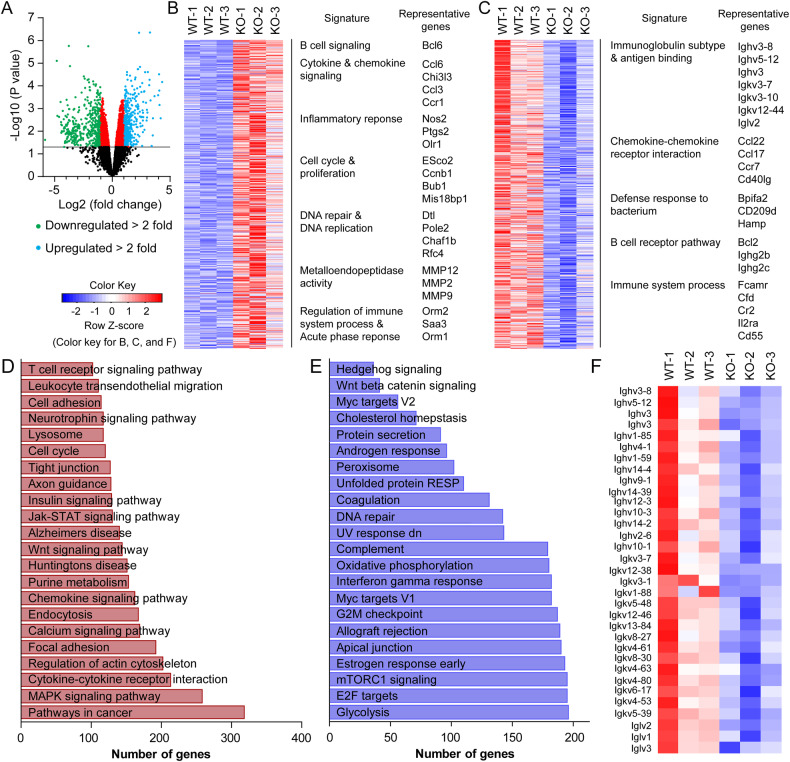
Microarray-based gene expression profiling of B-cell lymphomas in *Fbw7*^*ΔEC*^ mice. **A**–**C** Gene expression data was obtained using an Affymetrix GeneChip Mouse Gene 2.0 ST Array and mRNAs isolated from mesenteric lymph nodes (mLNs) from lymphoma-bearing *Fbw7*^*ΔEC*^ mice and normal mLNs from WT mice (*n* = 3 biological replicates per group). Volcano plot (**A**) and heatmap of genes significantly altered (*p* < 0.05) by twofold (upregulated genes in **B** and downregulated genes in **C**) in lymphoma**-**bearing mLNs from *Fbw7*^*ΔEC*^ mice compared with normal mLNs from WT mice. **D**, **E** Gene set enrichment analysis (GSEA) was used to identify biological terms, pathways, and processes that are coordinately up- or downregulated in lymphoma-bearing mLNs from *Fbw7*^*ΔEC*^ mice compared with WT mLNs. GSEA analysis using KEGG (**D**) and hallmark (**E**) gene sets. **F** Heatmap showing some significantly downregulated immunoglobulin heavy and light chain variable genes in lymphoma-bearing mLNs from *Fbw7*^*ΔEC*^ mice compared with normal mLNs from WT mice.

To further characterize these B-cell lymphomas, we compared gene expression profiles of mesenteric lymph nodes (mLNs) from lymphoma-bearing *Fbw7*^*ΔEC*^ mice and normal mLNs from WT mice using an Affymetrix^®^ Genechip^TM^ mouse gene array. Analysis of the relative average expression of all detected genes revealed that these B-cell lymphomas had distinct patterns of gene expression (Fig. 2A–C). Gene set enrichment analysis showed that significantly altered genes are primarily cancer-associated and inflammatory immune-associated genes in mLNs from lymphoma-bearing *Fbw7*^*ΔEC*^ mice (Fig. 2D, E), consistent with human DLBCL gene signatures [21]. Notably, numerous immunoglobulin heavy and light chain variable genes were dramatically decreased in lymphoma-bearing mLNs from *Fbw7*^*ΔEC*^ mice compared with normal mLNs from WT mice (Fig. 2F). Consistently, *Fbw7*^*ΔEC*^ mice showed significantly reduced levels of serum IgM and IgG (Fig. S8, Supporting Information), which is consistent with previous findings suggesting the low immunoglobulin levels in patients with non-Hodgkin lymphoma [22, 23]. Therefore, we provide an ideal animal model that resembles human DLBCL pathophysiology.

### Lymphoma B cells in *Fbw7*^*ΔEC*^ mice are endothelium-derived

**Fig. 3:**
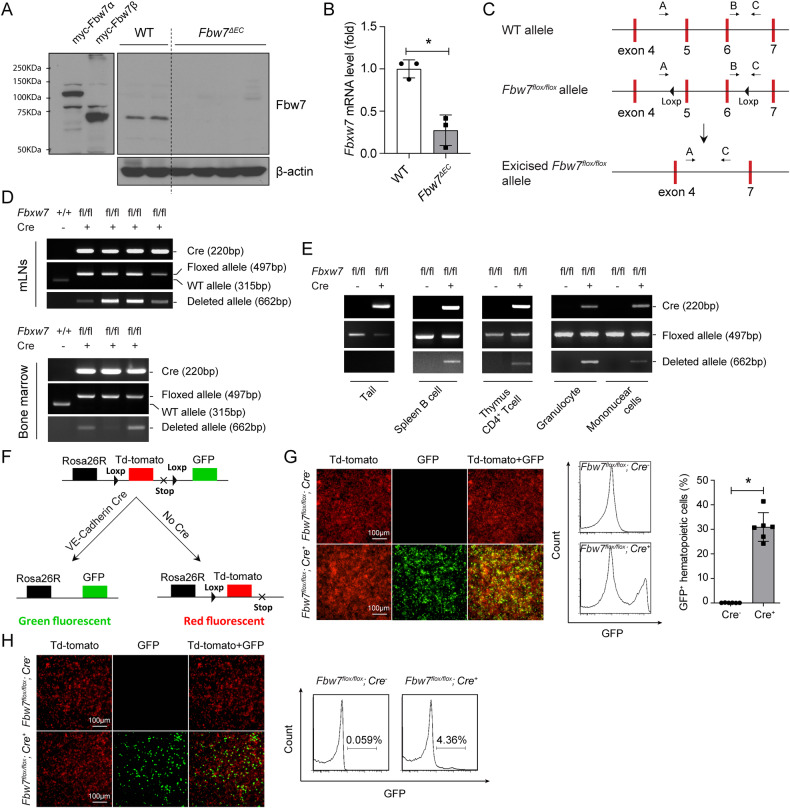
The lymphomas in *Fbw7*^*ΔEC*^ mice are *Fbxw7*-deficient. **A**, **B** Western blot and RT-PCR analysis of mLNs from WT and lymphoma-bearing *Fbw7*^*ΔEC*^ mice. Data represent mean ± SEM (*n* = 3 biological replicates per group). **C** Exons 4–7 of the *Fbxw7* locus before and after excision of the floxed exon, and primer locations. Primers B and C were used to detect WT and floxed alleles (315 and 497 bp, respectively). Primers A and C were used to detect deleted allele (662 bp). **D** Genotyping results for *Fbw7* in mLNs and bone marrow. **E** Genotyping results from indicated tissues. **F** *mTmG*^*+/-*^;*Fbw7*^*flox/flox*^;*VE-Cadherin-Cre*^*+*^ mouse generation. **G** Immunofluorescent (IF) images and flow cytometry analysis of bone marrow cells from *mTmG*^*+/-*^*;Fbw7*^*flox/flox*^*;VE-Cadherin-Cre*^*+*^ and *mTmG*^*+/-*^*;Fbw7*^*flox/flox*^*;VE-Cadherin-Cre*^*-*^ mice. Data represent mean ± SEM (*n* = 6 biological replicates per group). **H** IF of mononuclear cells and flow cytometry analysis of blood-derived B cells from *mTmG*^*+/-*^*;Fbw7*^*flox/flox*^*;VE-Cadherin-Cre*^*+*^ and *mTmG*^*+/-*^*;Fbw7*^*flox/flox*^*;VE-Cadherin-Cre*^*-*^ mice. *P* values were determined using the student’s *t*-test (**B**, **G**). For all panels, *p < 0.05.

To gain insight into the mechanism underlying spontaneous B-cell lymphomagenesis in *Fbw7*^*ΔEC*^ mice, we first assessed Fbw7 protein expression in lymphoma tissues from *Fbw7*^*ΔEC*^ mice. Loss of Fbw7 was detected in lymphoma-bearing mLNs from *Fbw7*^*ΔEC*^ mice, compared with normal mLNs from WT mice, where Fbw7 expression was intact (Fig. 3A). Quantitative RT-PCR analysis further confirmed decreased mRNA levels of *Fbxw7* in lymphoma tissues from *Fbw7*^*ΔEC*^ mice (Fig. 3B). Using genotyping (Fig. 3C) [24], we found that deletion of the *Fbxw7* locus was detected in several lymphoid tissues, including mLNs and bone marrow from lymphoma-bearing *Fbw7*^*ΔEC*^ mice (Fig. 3D). Furthermore, we found that B cells from the spleen, T cells from the thymus, and granulocytes and mononuclear cells from the blood also exhibited *Fbxw7* locus deletion (Fig. 3E). These observations confirm that the lymphomas in *Fbw7*^*ΔEC*^ mice were *Fbxw7*-deficient.

**Fig. 4:**
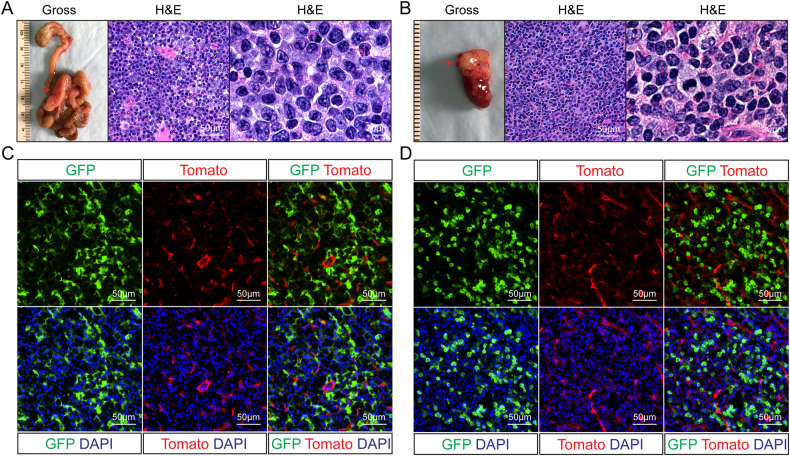
Lymphoma B cells in *Fbw7*^*ΔEC*^ mice are endothelium-derived. **A**, **B** Macroscopic images and H&E staining of mLNs (**A**) and thymus (**B**) from lymphoma-bearing *mTmG*^*+/-*^*;Fbw7*^*flox/flox*^*;VE-Cadherin-Cre*^*+*^ mice. **C**, **D** Immunofluorescent (IF) images of mLNs (**C**) and thymus (**D**) from lymphoma-bearing *mTmG*^*+/-*^*;Fbw7*^*flox/flox*^*;VE-Cadherin-Cre*^*+*^ mice.

To determine the origin of lymphoma B cells in *Fbw7*^*ΔEC*^ mice, we generated mice to perform lineage tracing: triple-transgenic *Fbw7*^*flox/flox*^*;VE-Cadherin-Cre;ROSA*^*mT/mG*^ mice (Fig. 3F). Using this model, we found that 31.5% of hematopoietic cells (CD45^+^ cells) in the bone marrow were derived from VE-Cadherin^+^ cells (Fig. 3G). Furthermore, we found that over 4% of B cells are also derived from VE-Cadherin^+^ cells (Fig. 3H). Most importantly, we found that the lymphoma tissues developed in *Fbw7*^*flox/flox*^*;VE-Cadherin-Cre;ROSA*^*mT/mG*^ mice displayed strong GFP signal (Fig. 4A–D), directly suggesting that the lymphoma B cells from *Fbw7*^*ΔEC*^ mice are endothelium-derived.

### BCL6 is a novel and specific FBW7 substrate in B cells

**Fig. 5:**
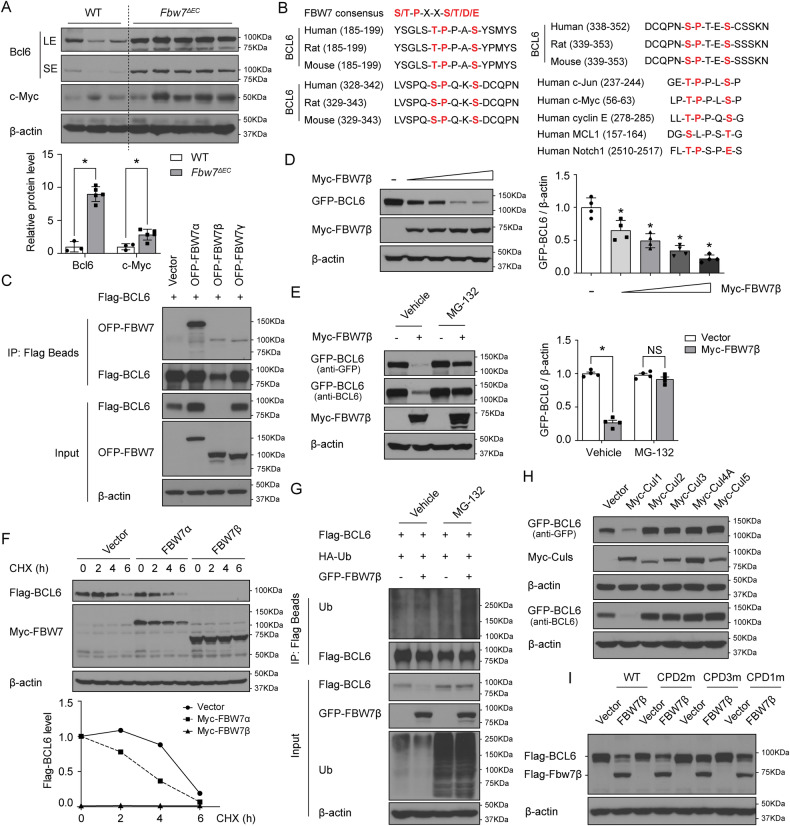
BCL6 is a novel and specific FBW7 substrate in B cells. **A** Western blot analysis for the indicated proteins in normal lymph nodes from WT mice and lymphoma-bearing lymph nodes from *Fbw7*^*ΔEC*^ mice. LE long exposure, SE short exposure. Data represent mean ± SEM (*n* = 3 for WT and *n* = 5 for *Fbw7*^*ΔEC*^ mice). **B** FBW7 consensus sequence (CPD) and alignment of BCL6 phosphodegron sequences recognized by FBW7 in different species to CPD motifs in indicated FBW7 substrates. **C** Pull-down analysis of exogenous Flag-BCL6 and OFP-FBW7 (α, β, or ɤ) in HEK-293T cells. **D** Western blot and quantification illustrating levels of GFP-BCL6 following the introduction of increasing amounts of Myc-FBW7β in HEK-293T cells. Data represent mean ± SEM (*n* = 4 biological replicates per group). **E** Western blot and quantification showing levels of indicated proteins after treatment with MG-132 (10 µM) in HEK-293T cells for 8 h. Data represent mean ± SEM (*n* = 4 biological replicates per group). **F** Western blot showing levels of indicated proteins in HEK-293T cells expressing Myc-FBW7α, Myc-FBW7β, or PCDNA following cycloheximide (CHX, 20 µg/ml) treatment for indicated times. **G** Pull-down analysis illustrating Flag-BCL6 ubiquitination in the presence of GFP-FBW7 in HEK-293T cells. **H** Western blot analysis of indicated proteins in HEK-293T cells in the presence of Myc-tagged Cullin proteins (Cul1, 2, 3, 4A, or 5). **I** Western blot analysis of indicated proteins in the presence of Flag-FBW7β and indicated mutants in HEK-293T cells: Flag-BCL6/T190A/S194A (CPD1m), Flag-BCL6/S333A/S337A (CPD2m), or Flag-BCL6/S343A/S347A (CPD3m). All western blots were normalized to β-actin. *P* values were determined using student’s *t*-test (**A**, **E**) or one-way ANOVA with Tukey’s multiple comparisons test (**D**). For all panels, *p < 0.05; NS indicates not significant.

Next, we explored the molecular mechanism by which Fbw7 deficiency induced B-cell lymphomagenesis. Consistent with our immunohistochemistry findings (Table S1 and Fig. S7, Supporting Information), elevated Bcl6 and c-Myc expression was observed in lymphoma-bearing mLNs from *Fbw7*^*ΔEC*^ mice, compared with normal mLNs from WT mice (Fig. 5A). As BCL6 is a proto-oncogene that is strongly implicated in the pathogenesis of human DLBCL [25–27], we hypothesized that BCL6 might be a novel substrate of FBW7 in B cells. As would be expected in canonical FBW7 substrates, three evolutionally conserved FBW7-recognizable degrons were found in the human BCL6 protein sequence (Fig. 5B). Although BCL6 bound to all three isoforms of FBW7 (FBW7α, β, and ɤ), only expression of FBW7β specifically and dramatically decreased BCL6 expression (Fig. 5C) in a dose-dependent manner (Fig. 5D). Cancer-associated mutant forms of FBW7β (R385H and R385C) corresponding to FBW7α (R465H and R465C) [11, 28] displayed a reduced ability to promote BCL6 degradation (Fig. S9A, B, Supporting Information). Moreover, FBW7β-mediated BCL6 elimination was abolished following treatment with the 26 S proteasome inhibitor MG-132 (Fig. 5E). These results suggest that FBW7β is the major isoform of FBW7 that regulates BCL6 protein level through the ubiquitin-proteasome pathway.

To further explore the role of FBW7β in modulating BCL6 stability, we examined BCL6 half-life in HEK-293T cells treated with the protein synthesis inhibitor cycloheximide. Co-expression of FBW7β with BCL6 significantly shortened the half-life of BCL6 (Fig. 5F). Consistent with a posttranslational mode of regulation, no alterations in *BCL6* mRNA levels were detected after overexpression of FBW7 in HEK-293T cells (Fig. S10, Supporting Information).

Since FBW7 directly binds to its substrate to promote its ubiquitination and degradation [29], we next determined whether FBW7 could directly stimulate BCL6 ubiquitination in vitro. As expected, overexpression of FBW7β triggered polyubiquitination of BCL6 (Fig. 5G). Since FBW7 is the substrate recognition component of the E3 ubiquitin ligase SKP1-cullin-1-FBW7 complex, we next assayed if overexpression of Cullin affects BCL6 stability. Overexpression of Cullin 1, but no other Cullin family members, significantly decreased BCL6 protein abundance (Fig. 5H). When we mutated the three FBW7-recognizable degrons in BCL6, we found that the S343A/S347A mutant form of BCL6 was resistant to FBW7-mediated BCL6 degradation (Fig. 5I). These collective data suggest that the E3 ubiquitin ligase SKP1-cullin-1-FBW7 complex targets BCL6 for ubiquitination and degradation in a degron-dependent manner.

### FBW7 expression levels inversely correlate with BCL6 expression

**Fig. 6:**
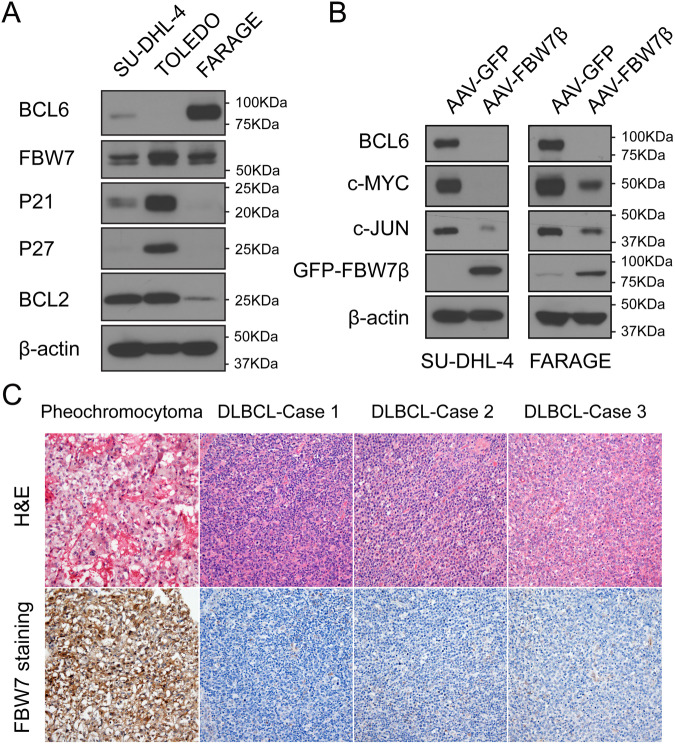
FBW7 expression levels inversely correlate with BCL6 expression. **A** Western blot analysis of BCL6, FBW7, P21, P27, BCL2, and β-actin in three different human diffuse large B-cell lymphoma cell lines. **B** Western blot analysis for the indicated proteins in cell lysates from SU-DHL-4 and Farage cells expressing AAV-GFP or AAV-GFP-FBW7β. **C** H&E staining and immunohistochemical analysis of FBW7 in a human DLBCL tissue array (US Biomax, LY121b). The pheochromocytoma samples were used as a control.

To validate the role of FBW7 in human B-cell lymphoma, we investigated the relationship of BCL6 and FBW7 in human B-cell lymphoma cell lines and found that expression of FBW7 inversely correlated with BCL6 expression (Fig. 6A). We next overexpressed FBW7β in the human B-cell lymphoma cell lines SU-DHL-4 and FARAGE, and found that BCL6 expression dramatically decreased following overexpression of FBW7β in both cell lines; levels of c-MYC and c-JUN, two well-characterized canonical FBW7 substrates, were used as positive controls (Fig. 6B). Strikingly, consistent with B-cell lymphomas in *Fbw7*^*ΔEC*^ mice, FBW7 expression was markedly decreased in clinical samples from patients with DLBCL (Fig. 6C). Taken together, these data further support that BCL6 is a novel substrate of FBW7 in the pathogenesis of lymphoid malignancy.

### B-cell lymphomagenesis in *Fbw7*^*ΔEC*^ mice is Bcl6-dependent

**Fig. 7:**
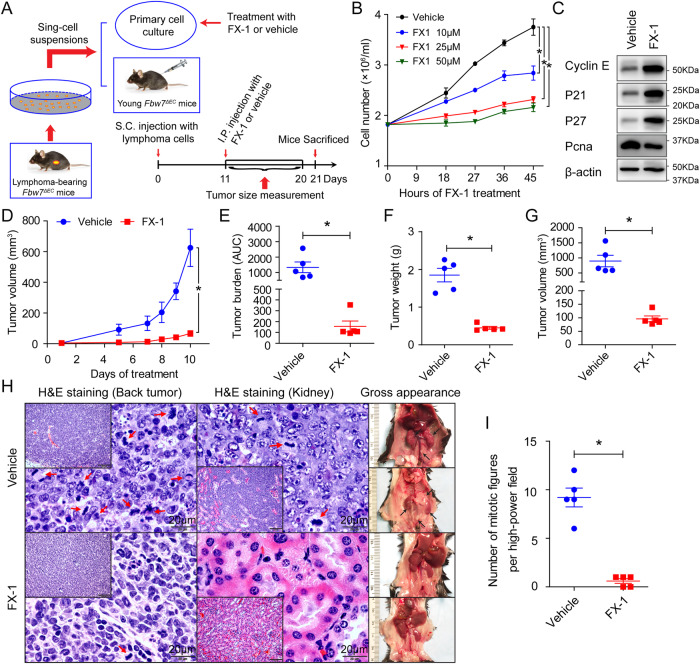
B-cell lymphomagenesis in *Fbw7*^*ΔEC*^ mice is Bcl6-dependent. **A** Schematic of tumor single-cell suspension preparation, primary DLBCL cell culture and treatment, and timeline for adoptive transfer experiments and FX-1 treatment. **B** Proliferation of primary DLBCL cells isolated from aged lymphoma-bearing *Fbw7*^*ΔEC*^ mice following FX-1 treatment for indicated doses and times. Data represent mean ± SEM (*n* = 4 biological replicates per group). **C** Western blot analysis for indicated proteins in primary *Fbw7*^*ΔEC*^ DLBCL cells following treatment with 10 µM FX-1 for 48 h. **D** Tumor growth by tumor volume during FX-1 treatment (*n* = 5 mice per group). **E** Tumor burden (area under the curve, AUC) for mice in **D**, calculated between initial and final volumes at day 10. **F**, **G** Tumor weight (**F**) and tumor volume (**G**) in mice upon sacrifice. Data represent mean ± SEM (*n* = 5 per group). **H** Left and middle, H&E staining of tissue sections from back tumors and kidneys of *Fbw7*^*ΔEC*^ mice following indicated treatments. Red arrows denote mitotic cells. Scale bars: 20 µm; 100 µm in inserts. Right, representative gross images of *Fbw7*^*ΔEC*^ mice treated with FX-1 or vehicle. Black arrows show organs infiltrated with lymphomas. **I** Quantification of mitotic figures in back tumors of indicated mice. Data represent mean ± SEM (*n* = 5 per group). *P* values were determined using ANOVA with Tukey’s post hoc test (**B**, **D**) and student’s *t*-test (**E**–**G**, and **I**). For all panels, *p < 0.05.

To validate that B-cell lymphoma development in *Fbw7*^*ΔEC*^ mice was BCL6-dependent, we performed adoptive transfer experiments using freshly isolated DLBCL cells from *Fbw7*^*ΔEC*^ mice. We found that fresh DLBCL cells from lymphoma-bearing *Fbw7*^*ΔEC*^ mice rapidly induced tumor growth in young healthy recipient *Fbw7*^*ΔEC*^ mice. Lymphoma cells showed clear and systemic metastatic capacity, and, in addition to subcutaneous tumors on the back, tumor deposits were found in different organs, including mesenteric and retroperitoneal LNs, thymus, liver, and kidney (Fig. S11, Supporting Information). Predominant expression of the B-cell marker B220 and weaker expression of the T-cell marker CD3e confirmed their B-cell origin, similar to that of the original transferred lymphoma (Fig. S11, Supporting Information). Next, we sought to determine whether lymphoma growth upon loss of Fbw7 is biologically dependent on Bcl6. To do so, we used the selective BCL6 inhibitor FX-1 [30] in in vitro and in vivo model systems (Fig. 7A). FX-1 significantly suppressed lymphoma cell growth in vitro (Fig. 7B, C), and, more importantly, mice treated with FX-1 had reduced tumor volume, tumor burden, and tumor weight compared with mice treated with vehicle control (Fig. 7D–G). Tumors from mice treated with FX-1 also displayed reduced cell proliferation and metastasis (Fig. 7H, I), suggesting that targeting BCL6 reduces this tumor growth mainly via induction of cell cycle arrest, which is consistent with the previous findings [31, 32].

DLBCL cells isolated from aged lymphoma-bearing *Fbw7*^*ΔEC*^ mice were next subcutaneously injected on the backs of immunocompromised NOD scid gamma (NSG) mice, which were subsequently treated with FX-1. Although subcutaneous back tumors did not develop in immunocompromised NSG mice, tumor deposits were found in several different organs, including the liver, kidney, spleen, and perirenal space (Fig. S12A, Supporting Information). Additionally, NSG mice treated with FX-1 showed significant attenuation of lymphoma growth and metastasis (Fig. S12A, B, Supporting Information). Together, these results suggest that B-cell lymphomagenesis in *Fbw7*^*ΔEC*^ mice is Bcl6-dependent.

## Discussion

In this present study, we discovered that conditional deletion of Fbw7 in VE-cadherin-positive endothelial cells promotes B-cell lymphomagenesis via upregulation of Bcl6 stability. Consistently, we demonstrated for the first time that Bcl6 is a novel substrate of FBW7, which directly interacts with Bcl6 and promotes its proteasomal degradation. Pharmacological disruption of Bcl6 abolishes Fbw7 deletion-induced B-cell lymphomagenesis. In addition, FBW7 expression is dramatically decreased in human DLBCL samples. Moreover, in human lymphoma cell lines, FBW7 expression levels are inversely correlated with BCL6 expression. Our work has provided new insights into cellular and molecular mechanisms underlying B-cell lymphomagenesis and suggests that genetic defects in endothelial cells can contribute to B-cell malignancies.

**Fig. 8:**
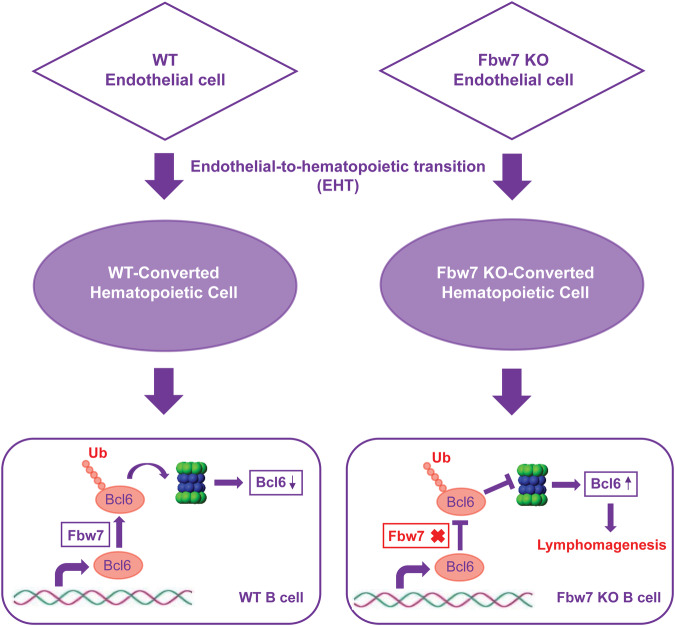
Schematic illustrating how endothelial Fbw7 deficiency contributes to B-cell lymphomagenesis. In this study, we propose that Fbw7-deficient B cells, which are derived from endothelial cells of *Fbw7*^*ΔEC*^ mice, exhibit Bcl6 accumulation and spontaneously develop DLBCLs.

Emerging evidence indicates that HSCs maintain a unique relationship with endothelial cells and are derived from a unique population of endothelial cells via EHT [4, 5, 7]. Endothelial cells are also an important microenvironmental component of the HSCs niche [33]. Therefore, we hypothesized that the spontaneous development of DLBCL in mice following the loss of Fbw7 could be due to either EHT or microenvironmental changes in the endothelium. Given that BCL6 is a transcriptional repressor that is relatively specific for germinal center B cells [27, 32, 34–36], our data suggest that Fbw7 deficiency-driven BCL6 accumulation in B cells, which are derived from endothelial cells, is the major mechanism behind B-cell lymphomagenesis in *Fbw7*^*ΔEC*^ mice (Fig. 8). Although Fbw7-deficient B cells compose only about 4% of the total B cells in young *Fbw7*^*ΔEC*^ mice, they are the “cancer seeds” that grow and prosper as age increases. Therefore, it takes a long duration of time for these few transformed B cells with Bcl6 accumulation to ultimately outnumber and take over normal B cells; this may explain why lymphomagenesis starts at 14 months of age in *Fbw7*^*ΔEC*^ mice. It is also worth noting that no other types of tumors or cancers were found in *Fbw7*^*ΔEC*^ mice, likely due to the fact that Bcl6 is exclusively expressed in germinal center B cells, but not in erythroid-myeloid progenitors and other hematopoietic cell types [27, 32, 34–36].

Endothelial cells produce all blood cells, including HSCs, during embryonic development [37, 38]. VE-cadherin is expressed in endothelial cells. The VE-cadherin Cre we used in this study is constitutive, i.e., non-inducible. The *Fbw7*^*flox/flox*^;*VE-cadherin-Cre* mice induce FBW7 deletion in all endothelial cells and blood cells, including HSC-independent and HSC-dependent hematopoietic cells. Therefore, whether the origin of this B-cell lymphoma is HSC-independent endothelial cells (directly from endothelial cells) or HSC-derived B cells remains unknown. Both inducible Cre^ERT2^ mice are warranted to validate the endothelial cell origins of DLBCL seen in *Fbw7*^*ΔEC*^ mice. Finally, complemental mouse models such as Fgd5-Cre^ERT2^, Vav1-Cre, CD19-Cre, or Mb1-Cre will be critical to determine which progenitor stage(s) are critical for lymphoma initiation by FBW7 deletion.

The ubiquitin ligase FBW7 targets a number of proto-oncoproteins for proteasomal degradation. Mutation or deletion of FBW7 has been strongly implicated in numerous T-cell and B-cell malignancies [11, 15, 28]. FBW7 regulates cellular apoptosis by targeting Mcl1 for ubiquitination and destruction and plays important roles in T-cell acute lymphoblastic leukemia [28]. *FBXW7* mutations reduce the binding of NOTCH1, leading to cleaved NOTCH1 accumulation and target gene activation in chronic lymphocytic leukemia [28]. A low expression level of FBW7 was associated with a poor outcome in non-GCB DLBC [15]. In the present study, we observed that mice with endothelial Fbw7 deletion spontaneously developed DLBCL, with FBW7 deficiency and Bcl6 accumulation in lymphoma tissue. Moreover, FBW7 reversely correlated with BCL6 in human B-cell lymphoma. Therefore, FBW7 acts as a tumor suppressor in DLBCL.

As BCL6 is a frequently activated oncogene in the pathogenesis of DLBCL [27], it has become a critical therapeutic target in DLBCL [30, 39]. Our findings demonstrate that BCL6 inhibition had anti-lymphoma activity in our DLBCL mouse model. This further supports our experimental evidence demonstrating a role for FBW7 in B-cell lymphomagenesis through regulation of BCL6 ubiquitination and stability, suggesting that FBW7/BCL6 could be a potential target in DLBCL therapy. Importantly, our animal model also provides a valuable preclinical platform for in vivo development and evaluation of novel therapeutic interventions for the treatment of DLBCL.

In summary, we provided the first evidence that loss of E3 ubiquitin ligase FBW7 in VE-cadherin positive endothelial cells instigates diffuse large B-cell lymphoma via upregulation of BCL6 stability.

## Experimental section

### Mice

*Vascular endothelial cadherin (VE-cadherin)-Cre* transgenic mice (Stock No. 006137) [40] and Fbw7-floxed (*Fbw7*^*flox/flox*^) mice (Stock No. 017563) [24] were purchased from The Jackson Laboratory. *VE-cadherin-Cre* mice were intercrossed with *Fbw7*^*flox/flox*^ mice to generate *Fbw7*^*flox/flox*^;*VE-cadherin-Cre* mice. *Fbw7*^*flox/flox*^;*VE-cadherin-Cre* mice were further bred with *ROSA*^*mT/mG*^ mice (The Jackson Laboratory, Stock No. 007576) to generate triple-transgenic *Fbw7*^*flox/flox*^*;VE-Cadherin-Cre*;*ROSA*^*mT/mG*^ mice. NSG mice (NOD.Cg-*Prkdc*^*scid*^;*Il2rg*^*tm1Wjl*^/SzJ, Stock No. 005557) were purchased from The Jackson Laboratory. Mice were housed in a controlled environment (22 °C, 12-h/12-h light/dark cycle) with free access to food and water. All mouse experimental protocols were approved by the Institutional Animal Care and Use Committee at Georgia State University and were in compliance with relevant ethical regulations. Genotyping for each strain was performed as described on The Jackson Laboratory website.

### Cell lines

HEK-293A and HEK-293T cells were maintained in Dulbecco’s Modified Eagle’s Medium (DMEM). SU-DHL-4 (ATCC^®^ CRL-2957^TM^), Toledo (ATCC^®^ CRL-2631^TM^), and Farage (ATCC^®^ CRL-2630^TM^) cells were grown in RPMI-1640 medium. All media were supplemented with 10% FCS, 100 U/ml penicillin, 100 U/ml streptomycin, and 2 mM l-glutamine.

### Tissue collection and processing

Tissue sections were stored at −80 °C for RNA extraction or western blotting. Tissue sections for pathological diagnosis or immunohistochemistry/immunofluorescence were fixed in 10% neutral buffered formalin, and embedded in paraffin or optimal cutting temperature (OCT) embedding material. Paraffin-embedded sections or OCT-embedded sections were cut at 5 or 8 μm thickness, respectively.

### Histology and immunohistochemistry

Paraffin-embedded tissue sections were stained with H & E, according to standard protocols. All images were recorded using an Olympus digital camera (Tokyo, Japan). For immunohistochemical staining, paraffin-embedded tissue sections were deparaffinized, rehydrated, and subjected to antigen retrieval. Endogenous peroxidase activity was blocked using 0.3% H_2_O_2_ for 20 min. Tissue sections were blocked with normal goat serum (Biogenex, HK112-9K) and incubated with primary antibodies against B220, CD3e, CD20, Bcl6, CD10, MUM1, and CD5. Anti-rabbit/mouse immunoglobulin G (DAKO, Cat# K5007) was used as a secondary antibody. The reaction was visualized using DAB (DAKO, Cat# K3468) and sections were counterstained with hematoxylin. Results were scored as 0 (0–25%, negative staining); 1 (25–50%, weakly positive staining); 2 (50–75%, moderately positive staining); or 3 (≥75%, strongly positive staining) according to the percentage of positive cells.

### Immunofluorescence

Immunofluorescence staining was performed as previously described [41–43]. Briefly, OCT-embedded sections were washed with phosphate-buffered saline (PBS), fixed with acetone at 4 °C for 15 min, and permeabilized with 0.2% Triton X-100 (Dow Chemical, Midland, MI, USA) for 10 min. After blocking with goat serum for 30 min, the sections were incubated with primary antibodies against Ki67 and B220 overnight. Secondary antibodies (Alexa Flour^®^ 555 goat anti-rabbit and Alexa Flour^®^ 488 goat anti-rat) were added for 1 h at 37 °C, followed by nuclear DNA staining using DAPI for 10 min. Fluorescence signals were evaluated using confocal microscopy (LSM 810, Zeiss, Oberkochen, Germany) or immunofluorescent microscopy.

### Isolation of B and T cells from spleen or thymus

The spleen or the thymus were removed from the mouse and transferred to a dish containing isolation buffer (PBS with 2% FBS + 2 mM EDTA). The tissues were further transferred to a cell strainer on top of a 50 ml tube and mashed into fine parts using the back of a syringe plunger. The filter was rinsed at regular intervals with an isolation buffer. The cell suspensions were filtered through a 70 µm nylon mesh and centrifuged at 300×*g* for 10 min at 4 °C. The cell pellets were collected, suspended with 5 ml/spleen of RBC lysis buffer (Qiagen, Cat# NC9670839), and incubated at room temperature for 5–10 min with occasional shaking. The cell suspensions were centrifuged, and the cell pellets were suspended in the appropriate buffer for use in the next step of the experimental procedure. B and T cells were further isolated using Dynabeads^TM^ Mouse CD43 (Thermo Fisher, Cat# 11422D) and Dynabeads^TM^ CD4 Positive Isolation Kit (Thermo Fisher, Cat# 11331D), respectively, according to the manufacturer’s instructions.

### Flow cytometry

Samples were obtained from blood, bone marrow, and spleen. Erythrocytes were lysed with RBC lysis buffer (Qiagen, Cat# NC9670839) for 10 min at room temperature. Single-cell suspensions were prepared in isolation buffer (PBS, 2% fetal bovine serum, 2 mM EDTA) and stained with anti-mouse CD19 (BD biosciences, Cat# 561738) or anti-mouse CD45 (BD biosciences, Cat# 559864) antibodies. Data were obtained using the LSRFortessa^TM^ X-20 cell analyzer (BD Biosciences).

### Preparation of tumor single-cell suspension

Tumor tissues from *Fbw7*^*ΔEC*^ mice were transferred into a 60 mm petri dish and washed with PBS. Transferred tissues were then cut into small pieces and digested in PBS containing 2 mg/ml collagenase D (Roche, Cat# 11088866001) and 100 µg/ml DNase I (Roche, Cat# 10104159001) for 1.5 h at 37 °C to obtain single-cell suspensions. Tumor cells were cultured in RPMI-1640 medium supplemented with 10% fetal calf serum (FCS), 100 U/ml penicillin, 100 U/ml streptomycin, and 2 mM l-glutamine, or resuspended in an isotonic solution of saline for injection.

### Adoptive transfer experiments

Freshly isolated tumor cells (1 × 10^7^) were injected subcutaneously on the backs of 6- to 8-week-old male *Fbw7*^*ΔEC*^ mice or immunocompromised NSG mice. For *Fbw7*^*ΔEC*^ mice, when back tumors reached a palpable size, mice were randomly assigned into an FX-1 (Targetmol, Cat# T4089) treatment group or vehicle control group. Mice were intraperitoneally injected with FX-1 (1 mg/day, reconstituted in PEG-400) or vehicle for 10 consecutive days. Tumor size was measured using an electronic digital caliper during the 10-day treatment. Tumor volume was calculated with the following formula: tumor volume (mm^3^) = (smallest diameter^2^ × largest diameter)/2.

### Constructs and mutagenesis

Plasmid information is presented in the Major Resources Tables in the Supplementary Data. Human FBW7β was cloned into an *Adeno-associated viral vector* modified from plasmid pAAV.CMV.PI.EGFP.WPRE.bGH. BCL6 mutant constructs were made by site-directed mutagenesis based on the pCXN2- BCL6 (Flag-tagged BCL6) wild-type construct using *KOD* Hot Start DNA polymerase (Xtreme, Millipore).

### Adeno-associated virus (AAV) packaging

AAV-2 was packaged using triple plasmid co-transfection in HEK-293T cells. Briefly, HEK-293T cells were transfected with AAV-FBW7β-acGFP (7 µg plasmid per 10 cm dish), an AAV helper plasmid (15 µg plasmid per 10 cm dish), and AAV serotype 2 (AAV-2, 7 µg plasmid per 10 cm dish). Around 48 h after transfection, cells were harvested.

### Western blotting

Total proteins were prepared from cultured cells or mouse tissues, and western blotting was performed, as briefly mentioned below. Proteins were quantified with a BCA assay (Pierce), separated by SDS-PAGE, and transferred to nitrocellulose membranes. Membranes were blocked with 5% non-fat milk dissolved in TBS-T at 37 °C for 1 h, and incubated with primary antibodies at 4 °C overnight. After incubation with horseradish peroxidase-conjugated secondary antibodies at 37 °C for 1 h, the membranes were washed 3 times with TBS-T and then visualized with chemiluminescent peroxidase reagents.

### RNA extraction and quantitative real-time PCR

Total RNA was extracted from mouse tissues using Trizol reagent (Invitrogen), according to the manufacturer’s instructions. Total RNA (1 μg) was reverse-transcribed into first-strand cDNA, and RT-PCR amplification was performed using SYBR Green dye and the CFX96 Touch^TM^ Real-time PCR Detection System (BIO-RAD). The primer sequences used for the detection of *Fbx7*, *Bcl6*, and *18S* are presented as follows: *Fbxw7* forward: 5’-GTGATAGAGCCCCAGTTCCA-3’; *Fbxw7* reverse: 5’-CCTCAGCCAAAATTCTCCAG-3’; *Bcl6* forward: 5’- TGATCGCTGCCAGGCCTCCTT-3’; *Bcl6* reverse: 5’- CTGGCCGATTGAACTGCGCTCC-3’; *18S* forward: 5’-GTCTGTGATGCCCTTAGATG-3’; *18S* reverse: 5’-AGCTTATGACCCGCACTTAC-3’. Relative mRNA expression was calculated using the comparative ΔΔCT method, and the resulting values were normalized to *18S* expression. PCR was performed in triplicate for each experiment. The results presented represent three independent experiments.

### Pull-down assay

Cells were washed with ice-cold PBS and lysed with IP lysis buffer (Pierce, Cat# 87787) supplemented with a proteinase inhibitor cocktail (Pierce, Cat# A32965) and phosphatase inhibitor (Thermo Fisher, Cat# 78428). Anti-FLAG M2 Affinity Gel (Sigma, Cat# A2220) was added to cell lysates and incubated overnight at 4 °C. The beads were then washed four times in a lysis buffer. After the last wash, FLAG-tagged proteins were eluted in an elution buffer and subjected to western blotting.

### ELISA

For the detection of IgM and IgG, plasma samples were collected from mice and immediately stored at −80 °C in a freezer until the day of the assay. An IgG/IgM mouse uncoated ELISA kit with plates was purchased from Invitrogen (Cat# 88-50400-22 & 88-50470-22). All reagents were provided in the ELISA kit and all procedures were performed according to the manufacturer’s instructions. Plates were read at 450 nm using a TECAN Infinite M1000 Pro microplate reader.

### Microarray analysis

RNA was extracted from sections of frozen biopsies using an RNeasy Mini Kit (Qiagen, CA, USA). Microarray-based gene expression profiling was performed using the Boston University Microarray and Sequencing Resource Core Facility. Mouse Gene 2.0 ST CEL files were normalized to produce gene-level expression values using the implementation of the Robust Multiarray Average (RMA) in the *affy* package included in the Bioconductor software suite (version 2.11) and an Entrez Gene-specific probeset mapping from the Molecular and Behavioral Neuroscience Institute (Brainarray) at the University of Michigan. Array quality was assessed by computing Relative Log Expression and Normalized Unscaled Standard Error using the *affyPLM* package. Principal Component Analysis was performed using the *prcomp* R function with expression values that had been normalized across all samples to a mean of zero and a standard deviation of one. Differential expression was assessed using the moderated (empirical Bayesian) *t*-test implemented using the *limma* package (i.e., creating simple linear models with *lmFit*, followed by empirical Bayesian adjustment with *eBayes*). Correction for multiple hypothesis testing was accomplished using the Benjamini-Hochberg false discovery rate. Human homologs of mouse genes were identified using HomoloGene. All microarray analyses were performed using the R environment for statistical computing.

### Gene set enrichment analysis (GSEA)

GSEA was used to identify biological terms, pathways, and processes that were coordinately up- or downregulated within each pairwise comparison. The Entrez Gene identifiers of human homologs of the genes interrogated by the array were ranked according to the *t* statistic computed using a knockout vs WT comparison. Any mouse genes with multiple human homologs (or vice versa) were removed prior to ranking, so that the ranked list represented only those human genes that match exactly one mouse gene. This ranked list was then used to perform pre-ranked GSEA analyses (default parameters with random seed 1234) using the Entrez Gene versions of the Hallmark, Biocarta, KEGG, Reactome, Gene Ontology, and transcription factor and microRNA motif gene sets obtained from the Molecular Signatures Database (MSigDB), version 6.0.

### Statistical analysis

Statistical analyses were performed using GraphPad Prism 5.0 software. Unpaired two-tailed Student’s *t*-tests were used to calculate significant differences between the two groups. Multiple comparison correction analysis was performed using one-way ANOVA with Tukey’s post hoc HSD test. A log-rank (Mantel–Cox) test was used to analyze Kaplan–Meier curves. Fisher’s exact test was applied to test for association between categorical variables. *P* values were considered significant when *P* < 0.05.

## Supplementary information

Supplementary Material-1

original data files

Reproducibility checklist

## Data Availability

All data is available in the main text or the supplementary files.
