# Supplementary material for: Selective deletion of E3 ubiquitin ligase FBW7 in VE-cadherin-positive cells instigates diffuse large B-cell lymphoma in mice in vivo
Source: Cell Death Dis. 2024 Mar 14;15(3):212. doi: 10.1038/s41419-024-06597-7 (PMC10940678; doi:10.1038/s41419-024-06597-7)
Supplement: Supplementary file 1 — Supplementary Material-1 [file 41419_2024_6597_MOESM1_ESM.docx]

**Supplemental Figures**


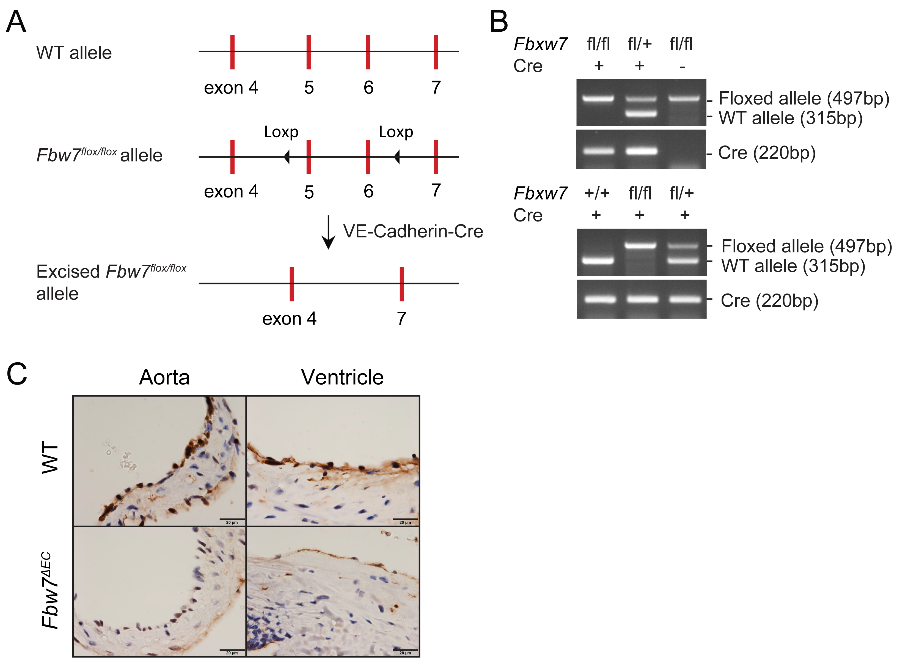


**Figure S1.** **Generation of *Fbw7^ΔEC^* mice**

(A) Schematic diagram of the transgenic mice used to generate *Fbw7^ΔEC^* mice.

(B) Genotype analysis for presence of the *Fbw7^ΔEC^* allele.

(C) Immunohistochemical staining for Fbw7 in aorta and heart tissue sections from WT and *Fbw7^ΔEC^* mice. Scale bars: 20 µm.


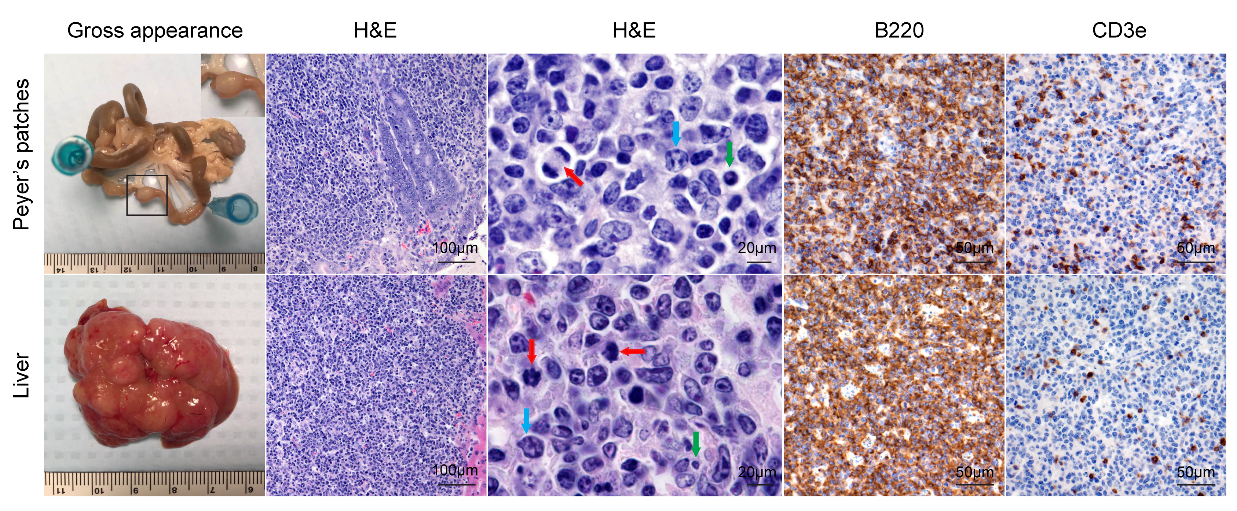


**Figure S2.** **Spontaneous development of B-cell lymphoma in *Fbw7^ΔEC^* mice**

Macroscopic images, H&E staining, and immunohistochemistry analysis for B220 (a marker of B cells) and CD3e (a marker of T cells) in lymphoma tissue sections from Peyer’s patches and livers of *Fbw7^ΔEC^* mice. Blue and green arrows denote centroblasts and immunoblasts, respectively. Red arrows denote mitotic cells.


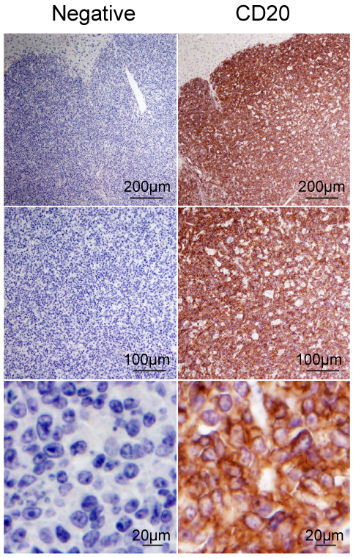


**Figure S3.** **Immunohistochemical staining of lymphoma tissues from *Fbw7^ΔEC^* mice**

Immunohistochemical staining for CD20 in lymphoma tissue sections of *Fbw7^ΔEC^* mice. As negative control, the CD20 antibody has been replaced by rabbit IgG.


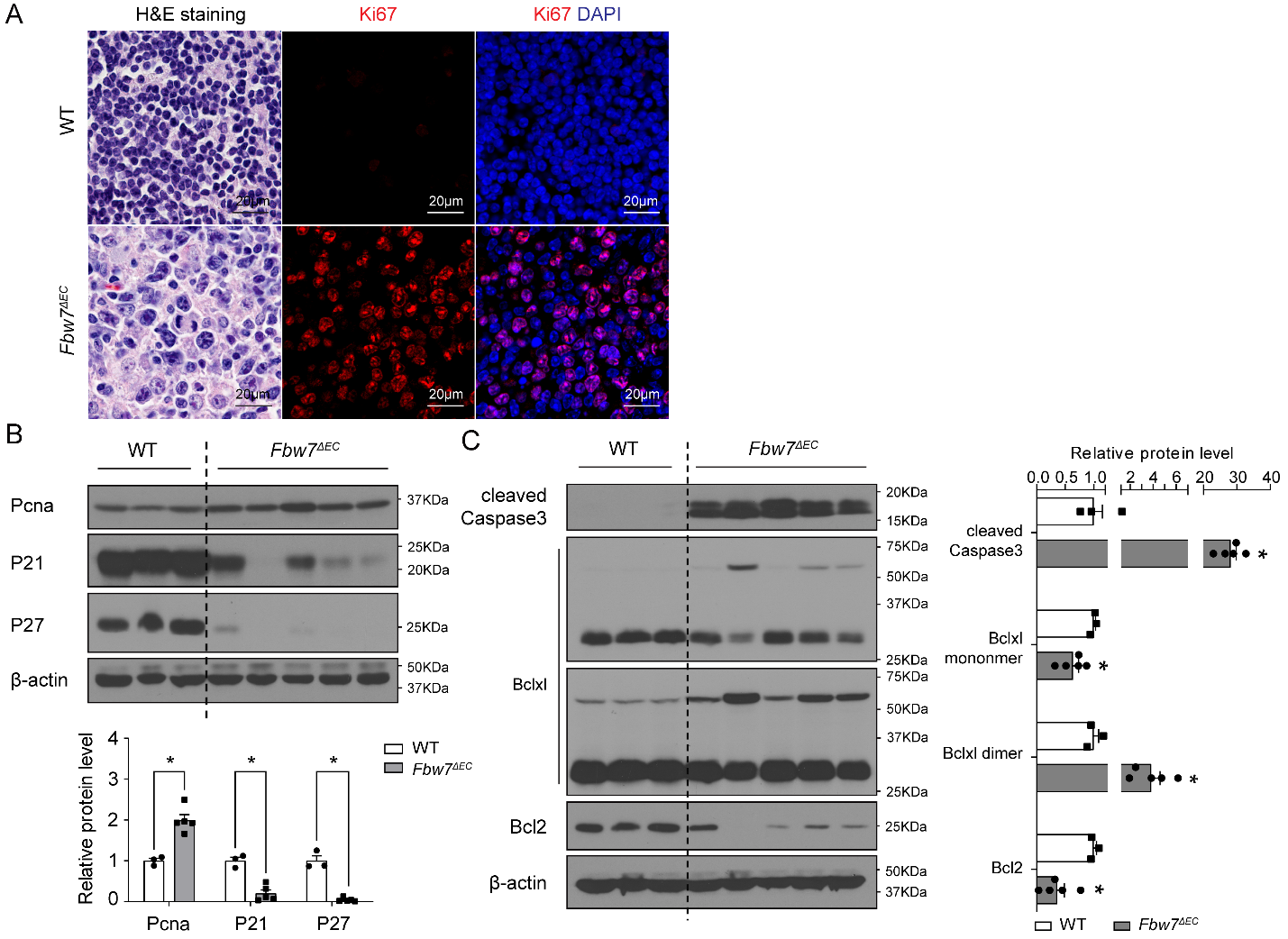


**Figure S4.** **B-cell lymphomas in *Fbw7^ΔEC^* mice display robust proliferation and increased apoptosis**

(A) Representative images of H&E staining and immunofluorescence staining for Ki67 (red) in lymphoma tissues from WT mice and lymphoma-bearing *Fbw7^ΔEC^* mice. Data represent mean ± SEM (n = 3 for WT and n = 5 for *Fbw7^ΔEC^* mice).

(B-C) Western blot analysis for Pcna, P21, P27, cleaved caspase-3, Bclxl, Bcl2, and β-actin in normal lymph nodes from WT mice and lymphoma-bearing lymph nodes from *Fbw7^ΔEC^* mice. Protein levels were normalized to β-actin levels. Data represent mean ± SEM (n = 3 for WT and n = 5 for *Fbw7^ΔEC^* mice). P values were determined using student’s t-test (B and C). For all panels, *p < 0.05.


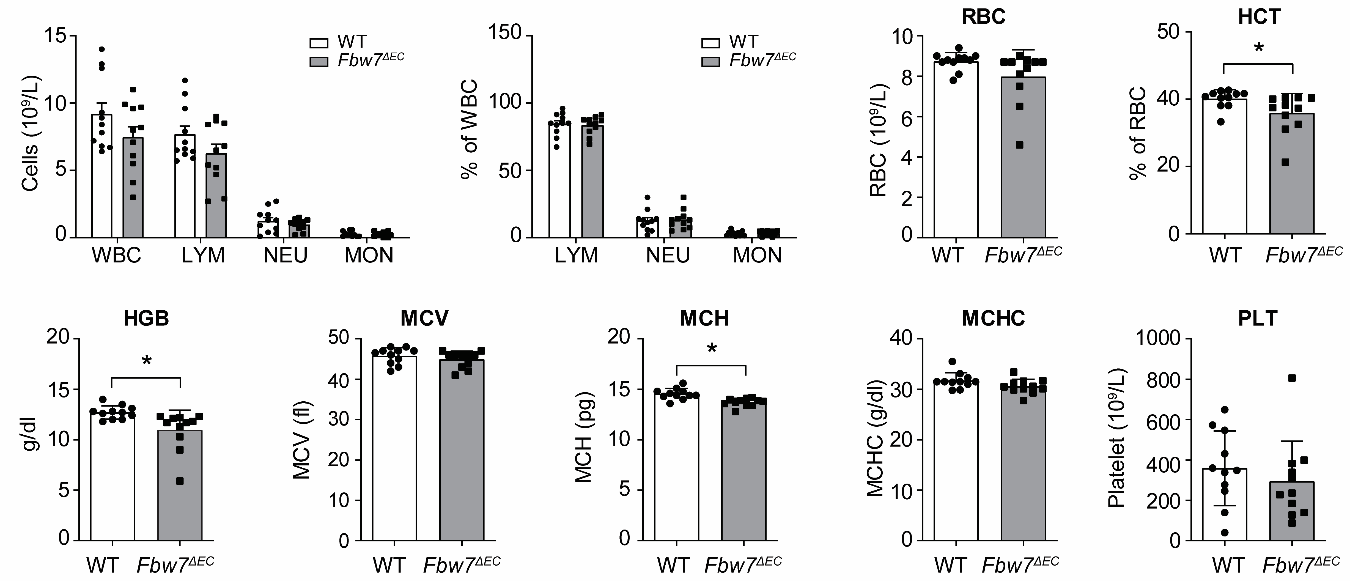


**Figure S5.** **Young *Fbw7^ΔEC^* mice showed comparable levels of white blood cells, lymphocytes, and monocytes as WT mice**

Whole blood counts of white blood cells (WBC), lymphocytes (LYM), neutrophils (NEU), monocytes (MON), red blood cells (RBC), hematocrit (HCT), hemoglobin (HGB), mean corpuscular volume (MCV), mean corpuscular hemoglobin (MCH), mean corpuscular hemoglobin concentration (MCHC), and platelets (PLT) from WT and *Fbw7^ΔEC^* mice aged 4-5 months. Data represent mean ± SEM (n = 11 biological replicates per group). P values were determined using student’s t-test. For all panels, *p < 0.05.


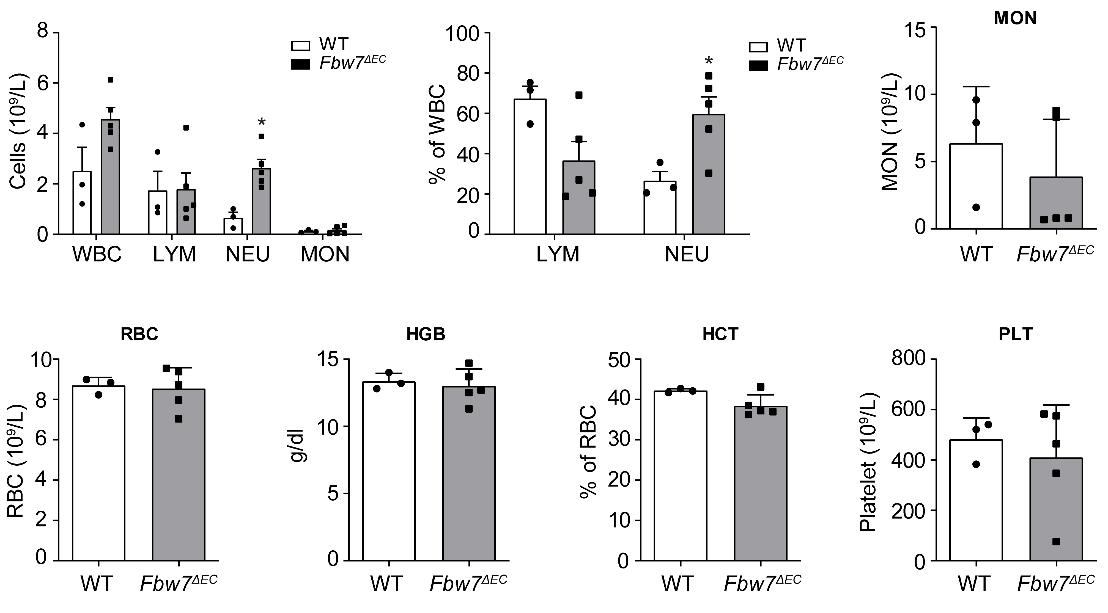


**Figure S6.** **Aged *Fbw7^ΔEC^* mice showed comparable levels of white blood cells, lymphocytes, and monocytes as WT mice**

Whole blood counts of white blood cells (WBC), lymphocytes (LYM), neutrophils (NEU), monocytes (MON), red blood cells (RBC), hemoglobin (HGB), hematocrit (HCT), and platelets (PLT) from WT and *Fbw7^ΔEC^* mice aged 16-18 months. Data represent mean ± SEM (n = 3 for WT and n = 5 for *Fbw7^ΔEC^* mice). P values were determined using student’s t-test. For all panels, *p < 0.05.

**
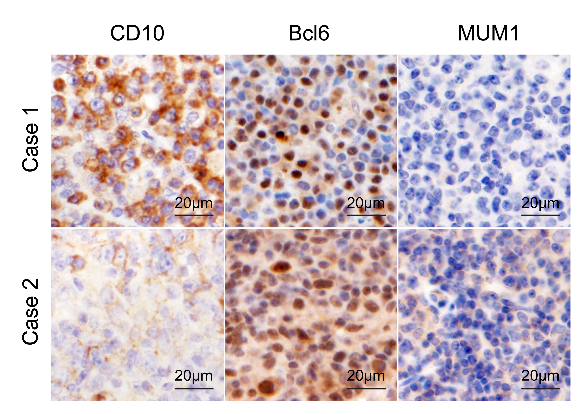
**

**Figure S7.** **Immunohistochemical staining of lymphoma tissues from *Fbw7^ΔEC^* mice**

Immunohistochemical staining for CD10, Bcl6 and MUM1 in lymphoma tissue sections of *Fbw7^ΔEC^* mice.


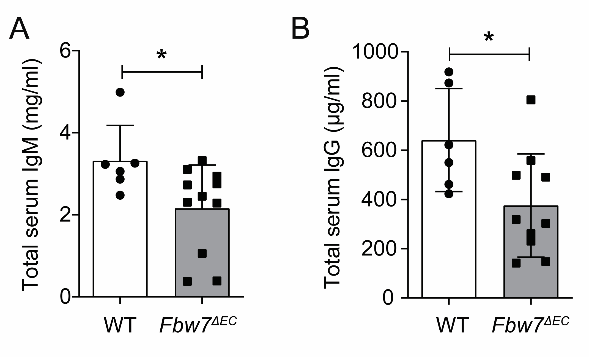


**Figure S8.** ***Fbw7^ΔEC^* mice showed decreased levels of serum IgM and IgG**

(A-B) ELISA analysis of total serum levels of IgM and IgG from WT and *Fbw7^ΔEC^* mice. Data represent mean ± SEM (n = 6 for WT and n = 10-11 for *Fbw7^ΔEC^* mice). P values were determined using student’s t-test (A and B). For all panels, *p < 0.05.


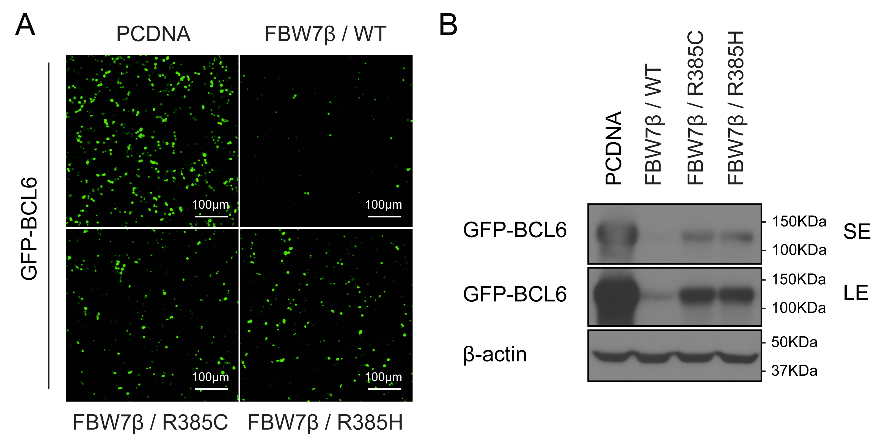


**Figure S9.** **Cancer-associated mutant forms of FBW7β have reduced ability to degrade BCL6**

(A-B) HEK-293T cells were transfected with GFP-BCL6 and FBW7β/WT, FBW7β/R385H, FBW7β/R385C, or a PCDNA control plasmid for 24 h. Immunofluorescent images showing GFP expression from cells expressing indicated mutants are shown in (A). Lysates from indicated transfected HEK-293T cells were prepared and analyzed by western blotting (B). SE, short exposure; LE, long exposure.


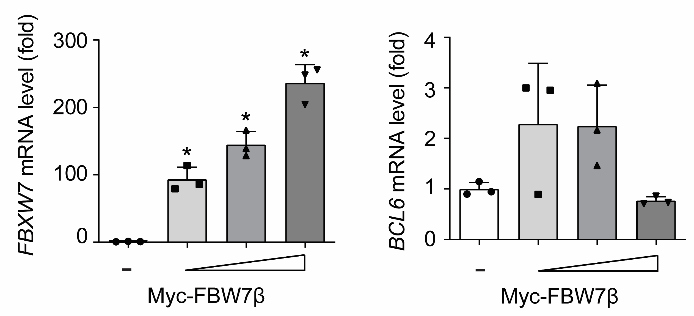


**Figure S10.** **FBW7 does not affect *BCL6* mRNA expression level**

HEK-293T cells were transfected with GFP-BCL6 and increasing amounts of myc-FBW7β for 24 h. Real-time PCR analysis was performed to examine the relative mRNA expression levels (normalized to 18S expression level) of *FBXW7* and *BCL6*. Data represent mean ± SEM (n = 3 biological replicates per group). P values were determined using one-way ANOVA with Tukey’s multiple comparisons test. For all panels, * P<0.05, compared with the group without myc-FBW7β co-transfection.


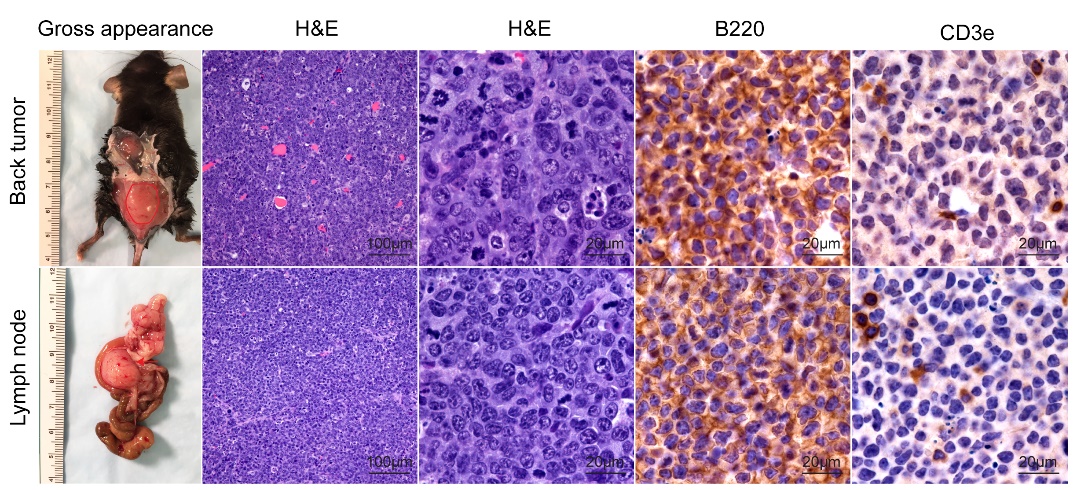


**Figure S11.** **Adoptive transfer of lymphoma cells from aged *Fbw7^ΔEC^* mice to young recipient *Fbw7^ΔEC^* mice**

Macroscopic images, H&E staining, and immunohistochemical analysis for B220 (a marker for B cells) and CD3e (a marker for T cells) in lymphoma tissue sections from back tumors and lymph nodes (LNs) of *Fbw7^ΔEC^* mice injected subcutaneously with lymphoma cells. Red lines trace the main tumor area. Red arrows denote enlarged mesenteric LNs.


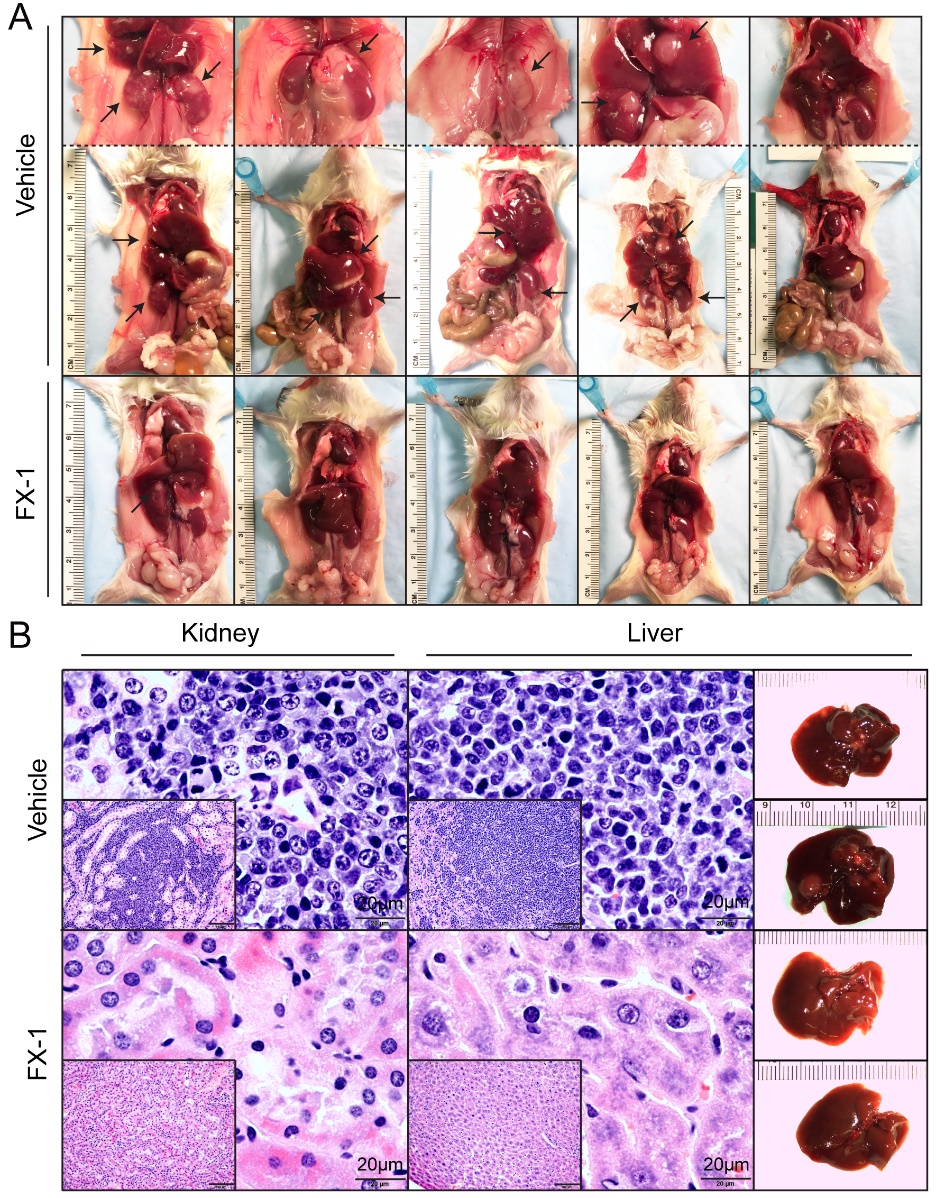


**Figure S12.** **The** **Bcl6 inhibitor FX-1 suppresses lymphoma development in NSG mice**

(A) Macroscopic images of NSG mice that were subcutaneously injected with freshly isolated lymphoma cells from aged lymphoma-bearing *Fbw7^ΔEC^* mice and treated with the Bcl6 inhibitor FX-1 or vehicle (n = 5 mice per group).

(B) H&E staining of tissue sections from kidneys and livers from NSG mice treated with FX-1 or vehicle, along with macroscopic images of livers from NSG mice treated with FX-1 or vehicle. Scale bars: 20 µm; 100 µm in small inserts.

**Supplementary Table 1. Lymphoma characteristics and tumor scoring.**

| # | Genotype | Pathological Diagnosis | Clinical Stage | B220 | CD3e | CD10 | MUM1 | Bcl6 | c-Myc | Ki67 index (%) |
| --- | --- | --- | --- | --- | --- | --- | --- | --- | --- | --- |
| 1 | *Fbw7^flox/flox^*;*VE-cadherin-Cre^+^* | GCB | IV | 3 | 1 | 3 | 0 | 3 | 1 | 35.93 ± 1.25 |
| 2 | *Fbw7^flox/+^*;*VE-cadherin-Cre^+^* | GCB | I | 3 | 0 | 0 | 0 | 2 | 3 | 68.98 ± 3.05 |
| 3 | *Fbw7^flox/flox^*;*VE-cadherin-Cre^+^* | GCB | III | 3 | 1 | 2 | 0 | 3 | 3 | 61.02 ± 2.31 |
| 4 | *Fbw7^flox/flox^*;*VE-cadherin-Cre^+^* | GCB | IV | 3 | 1 | 0 | 0 | 3 | 3 | 34.01 ± 3.01 |
| 5 | *Fbw7^flox/flox^*;*VE-cadherin-Cre^+^* | GCB | IV | 3 | 2 | 1 | 0 | 3 | 1 | 22.36 ± 5.15 |
| 6 | *Fbw7^flox/flox^*;*VE-cadherin-Cre^+^* | GCB | II | 3 | 2 | 2 | 1 | 3 | 3 | 39.96 ± 4.23 |
| 7 | *Fbw7^flox/flox^*;*VE-cadherin-Cre^+^* | GCB | IV | 3 | 2 | 0 | 0 | 3 | 2 | 26.25 ± 2.33 |
| 8 | *Fbw7^flox/flox^*;*VE-cadherin-Cre^+^* | GCB | IV | 3 | 2 | 3 | 1 | 3 | 2 | 33.31 ± 7.36 |
| 9 | *Fbw7^flox/flox^*;*VE-cadherin-Cre^+^* | GCB | II | 2 | 2 | 2 | 0 | 2 | 3 | 38.20 ± 2.62 |
| 10 | *Fbw7^flox/flox^*;*VE-cadherin-Cre^+^* | GCB | III | 2 | 2 | 2 | 1 | 3 | 1 | 19.92 ± 1.60 |
| 11 | *Fbw7^flox/+^*;*VE-cadherin-Cre^+^* | GCB | IV | 3 | 1 | 1 | 1 | 3 | 1 | 66.34 ± 8.56 |

GCB: germinal center B-cell like subtype of DLBCL.

Tumors were stained with indicated antibodies as described in Methods. IHC staining was scored as 0 (0-25%, negative staining); 1 (25-50%, weakly positive staining); 2 (50-75%, moderately positive staining); or 3 (≥75%, strongly positive staining) according to the percentage of positive cells.
