# Supplementary material for: Selective deletion of E3 ubiquitin ligase FBW7 in VE-cadherin-positive cells instigates diffuse large B-cell lymphoma in mice in vivo
Source: Cell Death Dis. 2024 Mar 14;15(3):212. doi: 10.1038/s41419-024-06597-7 (PMC10940678; doi:10.1038/s41419-024-06597-7)

Figure 3A

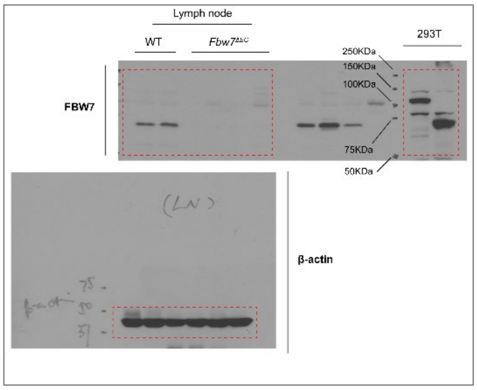

Figure 5A

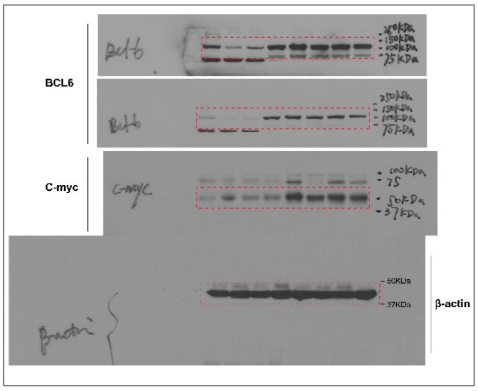

Figure 5C

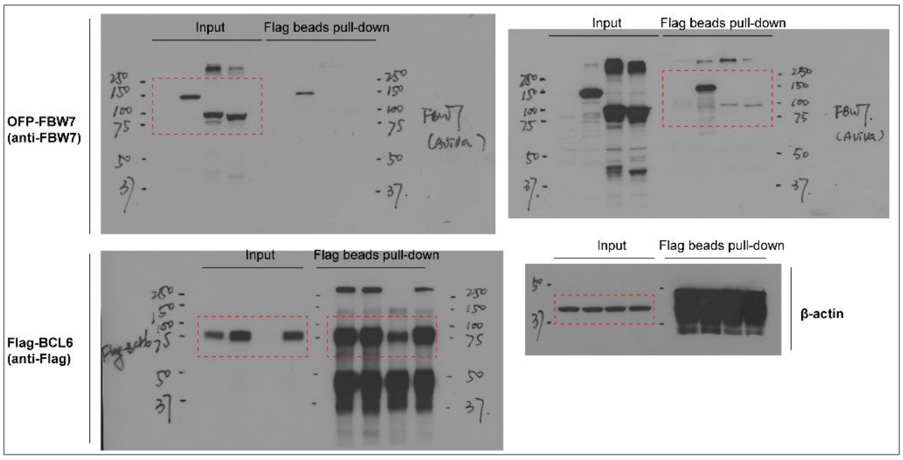

Figure 5D

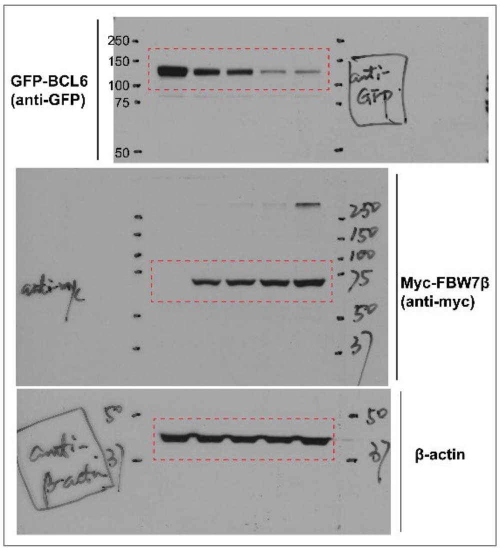

Figure 5E

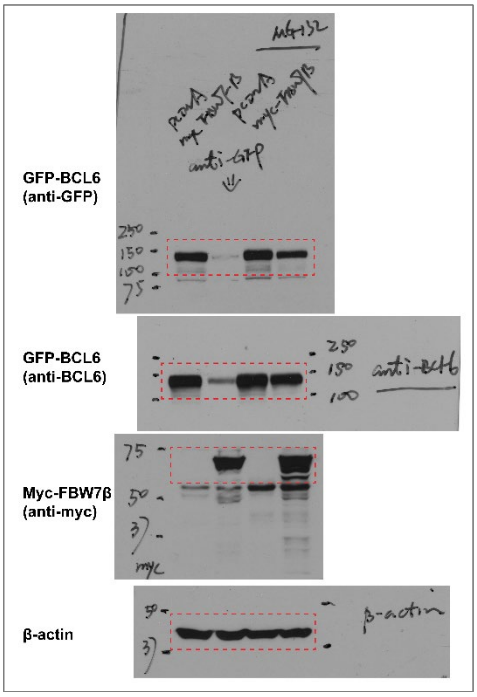

Figure 5F

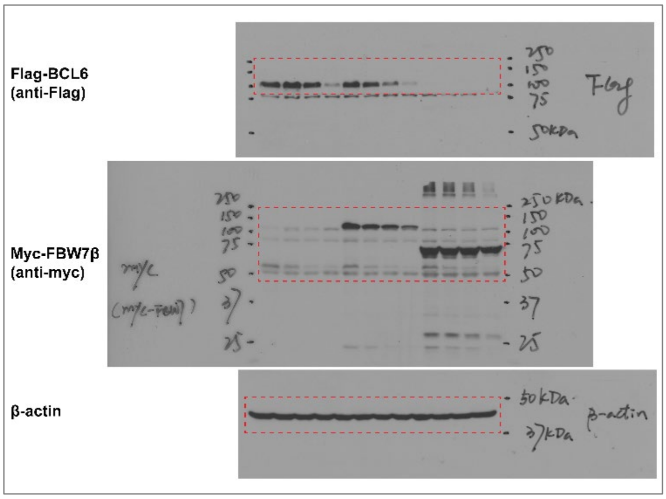

Figure 5G

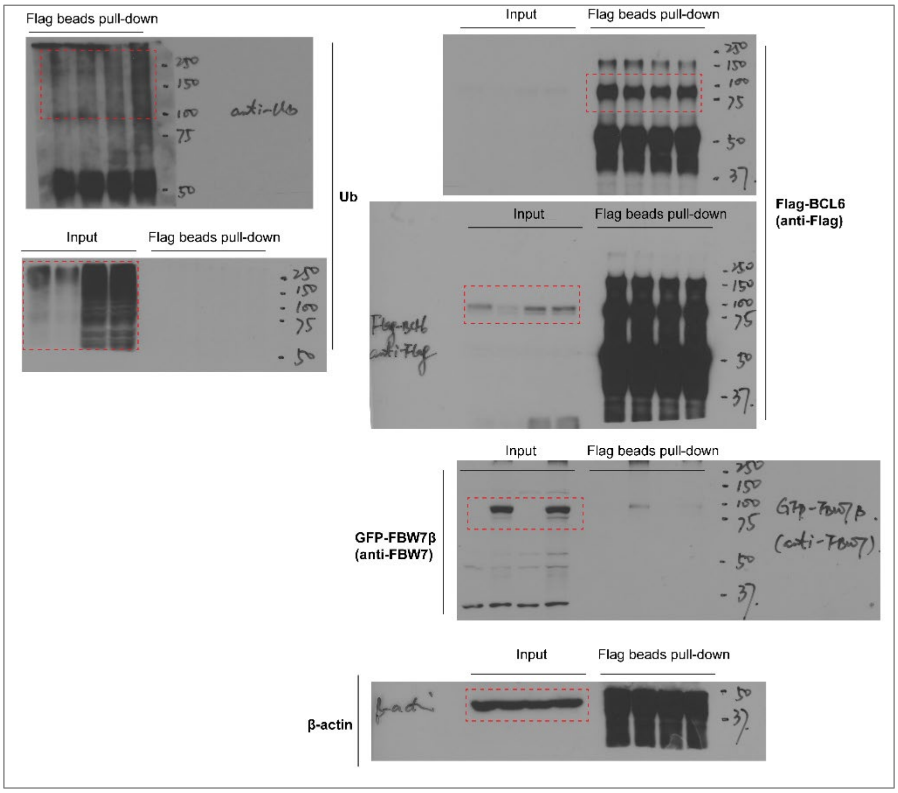

Figure 5H

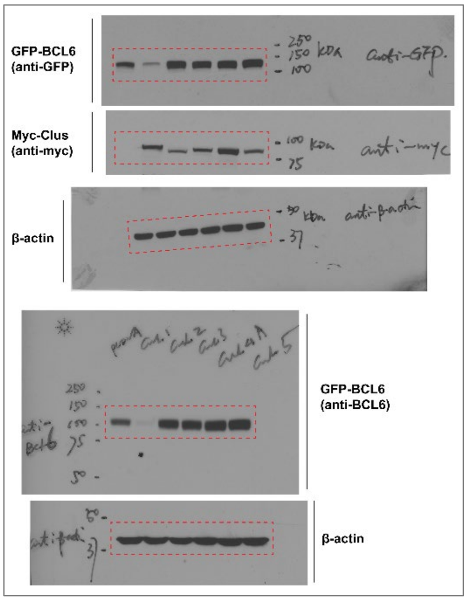

Figure 5I

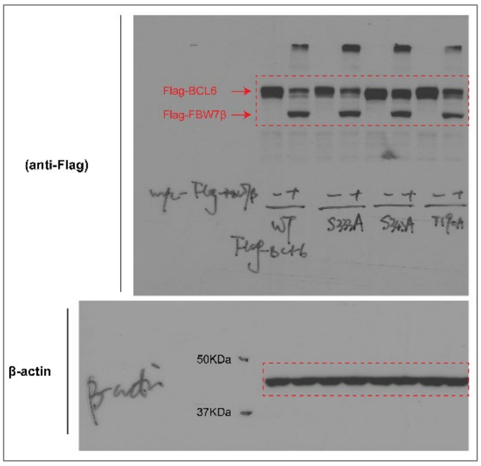

Figure 6A

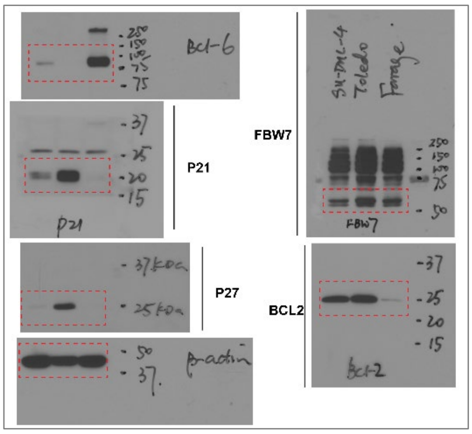

Figure 6B

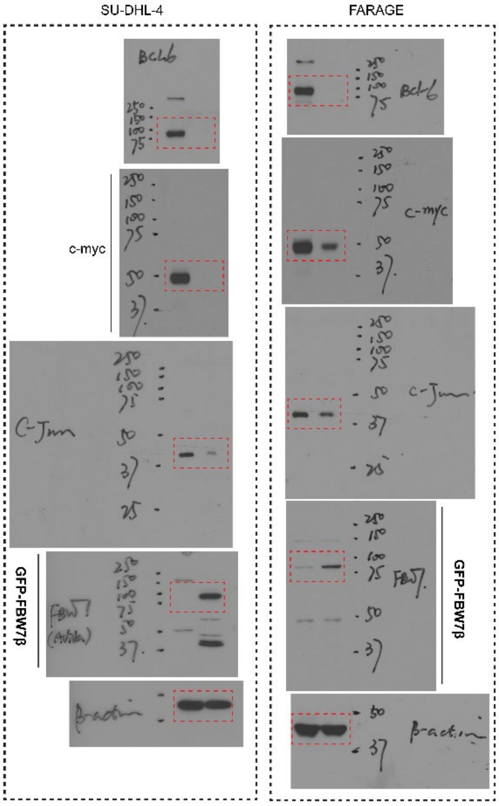

Figure 7C

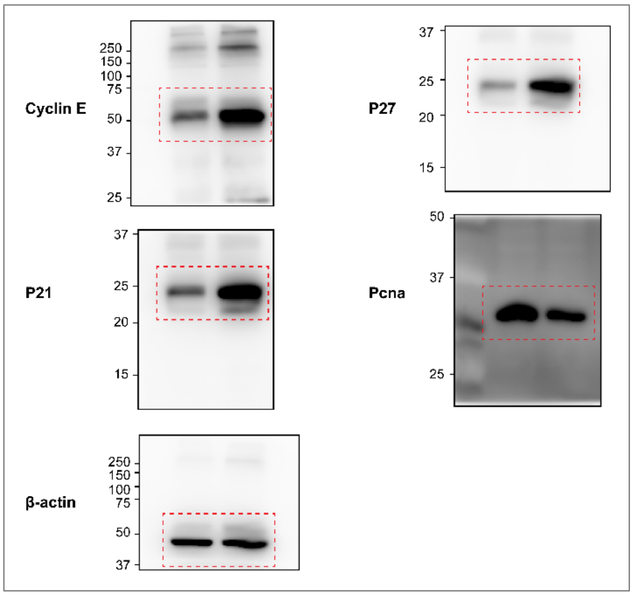
Cyclin E
250
150
100
75
50
37
25
P27
37
25
20
15
P21
37
25
20
15
Pcna
50
37
25
β-actin
250
150
100
75
50
37

Figure S4B

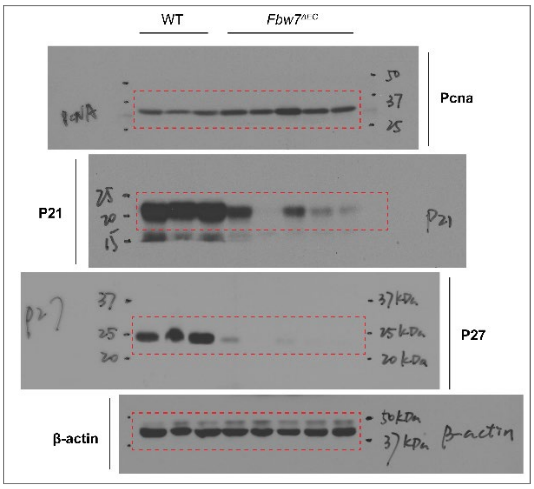

Figure S4C

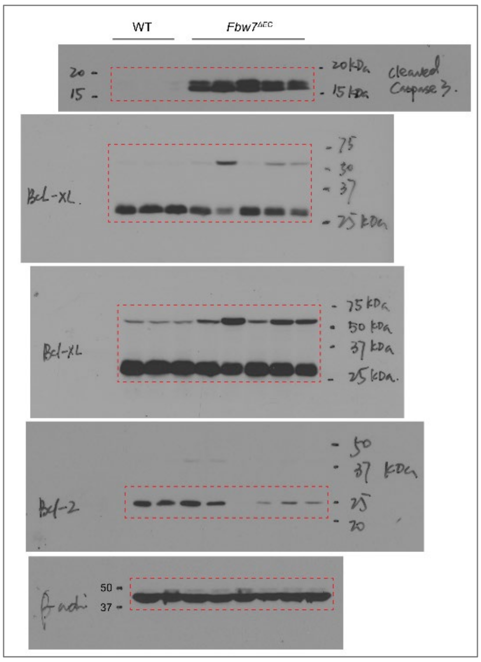

Figure S9B

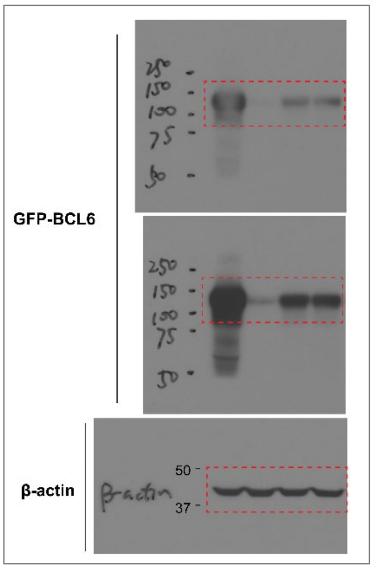

Supplement: Supplementary file 2 — original data files [file 41419_2024_6597_MOESM2_ESM.pdf]
